# Editorial: Meiosis: From Molecular Basis to Medicine

**DOI:** 10.3389/fcell.2021.812292

**Published:** 2021-12-01

**Authors:** Wei Li, Liangran Zhang, Akira Shinohara, Scott Keeney

**Affiliations:** ^1^ Guangzhou Women and Children’s Medical Center, Guangzhou Medical University, Guangzhou, China; ^2^ State Key Laboratory of Stem Cell and Reproductive Biology, Institute of Zoology, Chinese Academy of Sciences (CAS), Beijing, China; ^3^ Advanced Medical Research Institute, Shandong University, Jinan, China; ^4^ Institute for Protein Research, Osaka University, Suita, Japan; ^5^ Molecular Biology Program, Memorial Sloan Kettering Cancer Center, Howard Hughes Medical Institute, New York, NY, United States

**Keywords:** infertility, chromosome organization, chromosome movement, transcriptional regulation, post-translational modifications

## Introduction

Meiosis is a specialized cell division process in eukaryotes that produces haploid gametes from diploid cells by a single round of DNA replication followed with two successive rounds of chromosome segregation (meiosis I and meiosis II). Different from mitosis, meiosis has a prolonged prophase I characterized by many unique features including the formation of numerous programmed DNA double-strand breaks (DSBs) throughout the genome, most of which are repaired by recombination using the homologous chromosomes as templates. Meiotic recombination promotes the two-by-two pairing of homologous chromosomes and also disrupts the linkage relationships between segments of DNA along individual chromosomes to generate new combinations of alleles in offspring. Meanwhile, drastic chromatin remodeling and chromosome movements are needed to complete these processes, and these events are regulated at both transcriptional and posttranslational levels.

These processes ensure fertility, generate natural variation in populations, and provide the mechanistic basis for the rules of inheritance. Defects in meiosis are responsible for primary sterility, miscarriage, and congenital disorders. Thus, understanding the fundamental mechanisms underlying meiosis is not only very important to diagnosis of infertility and prevention of chromosomal birth defects, but also essential to the development of new strategies for improving animal breeding and crop production.

The 28 articles in this collection address many of the critical aspects of meiosis. They can be divided roughly into five categories: Chromosome Organization, Chromosome Movement, Transcription Regulation, Post-translational Modifications, and Diseases Related to Meiotic Defects.

### Overview

Thousands of genes have been reported to be involved in meiosis. A research article by Jiang et al. in this Research Topic developed “MeiosisOnline”, a publicly accessible, comprehensive database of known functional genes and potential candidates in meiosis (https://mcg.ustc.edu.cn/bsc/meiosis/index.html). A total of 2,052 genes were manually curated from literature resources and were classified into different categories. This resource provides updated and detailed information about both experimentally verified and predicted genes in meiosis (Jiang et al.).

While most meiotic processes occur similarly during gametogenesis in male and female mammals, significant differences have also been observed, a phenomenon known as sexual dimorphism in mammalian meiosis (Hunt and Hassold, 2002). There are substantial sex-specific differences within species with respect to meiosis-related chromatin reorganization, recombination, and tolerance for meiotic defects. A review by Hua and Liu in this Research Topic provided a comprehensive overview of genetically engineered mice that have been employed to study meiosis, with a particular focus on gene- and gametogenesis-related sexual dimorphism observed in these model animals.

### Chromosome Organization

A series of crucial events takes place during meiotic prophase I, including the formation and repair of DSBs, and the pairing, synapsis, and recombination between homologous chromosomes (Zickler and Kleckner, 1999; Handel and Schimenti, 2010). Meiotic DSB formation is catalyzed by the meiosis-specific transesterase SPO11 (Keeney et al., 1997; Milman et al., 2009), which is widely conserved across eukaryotic lineages (Hartung and Puchta, 2000). A review by Yadav and Claeys Bouuaert in this Research Topic described the mechanism of meiotic DSB formation and regulatory pathways in light of recent models. SPO11 has a DNA-binding domain but is not sequence-specific and requires the assistance of several other factors to induce programmed DSB production (Borde and de Massy, 2013). In mammalian cells, PRDM9, a protein with methyltransferase activity, is widely regarded as one of the most important regulators in this process (Baudat et al., 2010; Myers et al., 2010). Using a long-read sequencing approach, Alleva et al. expanded the catalog of known sequence variants within the *PRDM9* gene in human populations, and by mapping meiotic DSBs in testis, they found that small variations in PRDM9 can substantially alter the meiotic recombination landscape. These results demonstrated that minor PRDM9 variants may play an under-appreciated role in shaping patterns of human recombination (Alleva et al.).

After programmed DSB formation, single-stranded DNA near the programmed DSB site is covered by the RPA complex (RPA1/2/3) to protect it from DNA nuclease degradation, and the RPA-bound single strand is recognized by ATR (Harrison and Haber, 2006; Lovejoy and Cortez, 2009). ATM and ATR, as DNA damage-responsive kinases, can phosphorylate downstream effector kinases such as CHK1 and CHK2 (RAD53), thus playing an important role in DSB repair and homologous recombination during the prophase I (Subramanian and Hochwagen, 2014). A research article by Usui and Shinohara in this Research Topic examined how meiotic *Saccharomyces cerevisiae* cells activate Rad53 in response to exogenous DSBs and showed that this activation is dependent on an epigenetic mark, Dot1-dependent histone H3 lysine 79 methylation. This methylation becomes a scaffold for a Rad53 mediator, Rad9, which is an ortholog of mammalian 53BP1 (Usui and Shinohara). Interestingly, Rad9 is specific for exogenous damage, and is insensitive to meiotic DSBs. As an intermediate factor in DSB repair, BRCA1 can also be phosphorylated by ATM and ATR (Caestecker and Van de Walle, 2013), thereby activating the recruitment of BRCA2. BRCA2 and BRCA1 further promote recruitment of RAD51 (Scully et al., 1997; Lord and Ashworth, 2007) and DMC1 (Sharan et al., 2004; Martinez et al., 2016). A review by Li and Engebrecht summarizes work from mice and worms that has shed light on the role of BRCA1 and BRCA2 in meiosis.

Meiotic crossovers are formed via repair of DSBs. The proper number and placement of crossovers are vital to chromosome segregation (Mets and Meyer, 2009). A review by Pazhayam et al. in this Research Topic discussed the history of studies of crossover patterning, developments in the methods used in the field, and the current understanding of the interplay between patterning phenomena.

During meiosis, homologous chromosomes become juxtaposed along their lengths, stabilized by a proteinaceous scaffold known as the synaptonemal complex, consisting of a linear axial element for each homolog bridged by central region proteins (Moses, 1956; Page and Hawley, 2004; Costa et al., 2005; Bolcun-Filas et al., 2009; Schramm et al., 2011). A review by Grey and de Massy in this Research Topic summarized the different actors involved in axial element formation in *Saccharomyces cerevisiae* and mice. They also described the current knowledge of their localization patterns during prophase I, their functional interdependence, their roles in sister chromatid cohesion, formation of higher order loop-axis structure, homolog pairing before meiotic recombination, and recombination (Grey and de Massy). In human and zebrafish spermatocytes, homologous recombination and assembly of the synaptonemal complex initiate predominantly near telomeres (Saito et al., 2013; Pratto et al., 2014). A research article by Imai et al. in this Research Topic demonstrated that *Sycp1* is not required for peri-telomeric DSB formation but is necessary for the complete pairing of homologs during zebrafish meiosis.

In most mammals, chromosomal segments that do not form synaptonemal complex (for example, if there is no homolog present) undergo a transcriptional silencing process called meiotic silencing of unpaired chromatin (MSUC) (Turner, 2015). Meiotic sex chromosome inactivation (MSCI) is the process by which nonhomologous portions of the X and Y chromosomes of male mammals undergo MSUC during meiotic prophase I of spermatogenesis. MSCI is accompanied by formation of a special nuclear territory known as the sex body or XY body. A paper by Xu and Qiao in this Research Topic summarized recent publications on the mechanisms of MSCI and of liquid-liquid phase separation (LLPS), hypothesized a potential link between LLPS and the formation of sex bodies, and discussed the implications for future research.

### Chromosome Movement

Dramatic chromosome movements occur during meiotic prophase I in all organisms where this has been evaluated to date (Zickler and Kleckner, 1999; Petronczki et al., 2003; Alleva and Smolikove, 2017). These movements are driven by the attachment of telomeres through the nuclear envelope (NE) to cytoplasmic force-generating cytoskeleton structures via the LINC (linker of nucleoskeleton and cytoskeleton) complex (Crisp et al., 2006). In mouse, this involves LINC complex components SUN1, SUN2, KASH5, and telomeric adaptor proteins like TERB1, TERB2, MAJIN, SPDYA, and TRF1 (Tapley and Starr, 2013; Wang et al., 2018). Three articles in this Research Topic address this subject. A research article by Wang et al. found that SUN1 not only interacts with TERB1 but also more strongly with MAJIN. They also found that SUN1 interacts with SPDYA, an activator of CDK2. These findings provide the possible mechanism of SUN1, MAJIN, and SPDYA-CDK2 in promoting the telomere-NE attachment during meiosis (Wang et al.). Another research article in this Research Topic by González-Arranz et al. reveals that H2A.Z, a variant of the canonical H2A histone, is an additional LINC-associated factor that contributes to telomere-driven chromosome motion critical for error-free gametogenesis. Nozaki et al. developed a technique combining fluorescent repressor operator system (FROS) labeling with three-dimensional (3D) live-cell imaging at a high temporal resolution to define the detailed kinetics of mid-meiotic prophase I motion for a single telomere-proximal locus in budding yeast.

Accurate chromosome segregation during the two meiotic divisions relies on the attachments between spindle microtubules and kinetochores, multiprotein complexes that assemble on centromeres (McKim and Hawley, 1995). A review by Sato et al. in this Research Topic focuses on lessons from recent advancement in genetic and cytological studies of the fission yeast *Schizosaccharomyces pombe*. They discuss how chromosomes, the cytoskeleton, and cell cycle progression are organized and particularly how these differ in mitosis and meiosis.

In oocytes of many species, the assembly and organization of a specialized acentriolar spindle require the aid of multiple microtubule organizing centers (MTOCs), which contain essential pericentriolar materials (Mullen et al., 2019). A research article by Yin et al. reports that echinoderm microtubule-associated protein (EMAP)-like 1 (EML1), a member of the conserved EMAP family proteins, regulates acentriolar spindle formation and the progression to meiosis II in mammalian oocytes.

### Transcriptional Regulation of Meiosis

Unique transcription regulation is involved in the complex and highly organized events during meiosis. Technical advances in recent years, from the improvement of flow cytometry (FCM) to single-cell RNA-seq (scRNA-seq) approaches, allow accurate identification of cell heterogeneities and investigation of meiotic transcriptomes at a higher temporal resolution (Tang et al., 2009; Geisinger and Rodríguez-Casuriaga, 2017; Green et al., 2018).

Two articles in this Research Topic address this subject. A review by Geisinger et al. focuses on murine male meiosis and outlines the diversity of approaches and methodologies, shedding light on the transcriptomic landscape of the complex meiotic process. Particularly, they center on the controversy about gene expression during early meiotic prophase; the widespread existing gap between transcription and translation in meiotic cells; the expression patterns and potential roles of meiotic long noncoding RNAs; and the visualization of meiotic sex chromosome inactivation from the RNA-seq perspective (Geisinger et al.). Another review by Peng and Qiao compared bulk RNA-seq and scRNA-seq to show the advantages of scRNA-seq in meiosis studies. They also summarized scRNA-seq analysis methods and meiotic marker genes from spermatocytes and oocytes. Specifically, they emphasized the different features of two scRNA-seq protocols (Smart-seq2 and Drop-seq) in the context of meiosis studies and discussed their relative strengths and weaknesses.

Meiotic cells have complex transcriptomes, in that they express multiple mRNAs, long non-coding RNAs (lncRNAs), and small non-coding RNAs (sncRNAs) such as microRNAs (miRNAs) and PIWI-interacting RNAs (piRNAs) (Soumillon et al., 2013). These different classes of RNAs play important roles in meiosis and spermiogenesis (Goh et al., 2015; Perez and Lehner, 2019; Dai et al., 2020; Guo et al., 2020). A review by Li et al. in this Research Topic focuses on the current knowledge derived from studies of genetically engineered mouse mutants and summarizes pathways for the biogenesis and function of piRNAs.

RNA interference (RNAi) is an evolutionarily conserved cellular process involving double-stranded RNA (dsRNA) and regulating complementary RNA transcripts (Hannon, 2002). Dicer and Argonaute (Ago) are key proteins involved in this pathway (Carmell et al., 2002; Förstemann et al., 2007). Many studies have demonstrated that RNAi components play essential roles in meiotic processes including DNA repair and chromosome segregation (Burger and Gullerova, 2015; Gutbrod and Martienssen, 2020). A research article in this Research Topic by Girard et al. shows that in the filamentous Ascomycete *Sordaria macrospora*, genes encoding the two Dicer (Dcl1 and Dcl2) and two Argonaute (Sms2 and Qde2) proteins play critical roles for meiocyte formation, chromosome axis lengths, and crossover patterning.

### Regulation of Meiosis by Post-Translational Modifications

The acetylation and methylation of histones, as well as the phosphorylation, SUMOylation, and ubiquitination of other protein factors, play important roles during meiosis in the programmed formation and repair of DSBs, the pairing of homologous chromosomes, and the formation of crossovers (Zentner and Henikoff, 2013).

Histone acetylation was the first histone modification discovered to regulate gene transcription by fine-tuning chromatin accessibility (Lee et al., 1993). Histone acetyltransferases (HATs) and histone deacetylases (HDACs) are the main enzymes responsible for lysine acetylation and deacetylation of histones (Legube and Trouche, 2003; Wang et al., 2017). Histone acetylation has an important effect on the meiosis during spermatogenesis and oogenesis (Getun et al., 2017). A research article by Shi et al. in this Research Topic finds that the absence of histone H3 lysine 18 acetylation (H3K18ac) in *S. cerevisiae* impairs respiration, leading to reduced levels of Rim101 protein, which further upregulates Smp1 (a negative regulator of *IME1* transcription) and blocks the initiation of meiosis.

Histone methylation is perhaps the most well-studied histone modification, and it most frequently occurs on lysine and arginine residues of histones H3 and H4 (Zhang and Reinberg, 2001; Kouzarides, 2002). Methylation of lysine residues is catalyzed by HMTs (histone methyltransferases), including the SET-domain-containing protein family and the non-SET-domain proteins Dot1/DOT1L. Methylation of H3K4, H3K36, and H3K79 is associated with gene activation, whereas methylation of H3K9, H3K27, and H4K20 is generally thought to be involved in gene repression (Martin and Zhang, 2005). A research article by Dong et al. in this Research Topic demonstrates that PRMT5 regulates mouse spermatogonial stem cell development by modulating histone H3 lysine modifications.

Protein phosphorylation, which reversibly occurs mainly on serine, threonine, or tyrosine residues, is another well-studied post-translational modifications. Two articles in this Research Topic address this subject. A review by Kar and Hochwagen discussed common principles and provided detailed examples of how phosphorylation events are employed to ensure faithful passage of chromosomes from one generation to the next. Furthermore, a review by Lei et al. discussed recent discoveries and explored the role of PP2A-like protein, which regulates the dynamic equilibrium of protein phosphorylation and dephosphorylation during meiotic progression.

### Diseases Related to Meiotic Defects

Primary ovarian insufficiency (POI) is defined as ovarian follicle depletion or dysfunction before the age of 40. The etiology of POI is heterogeneous. Genetic defects, including monogenic mutations and chromosomal abnormalities (Qin et al., 2015; Jiao et al., 2017), found in approximately 20–25% of POI patients, are considered to be one of the main causes of POI, (Welt, 2008; Nelson, 2009). The review by Huang et al. focused on genes participating in meiotic homologous recombination and categorized the individual gene mutations identified in POI patients and the potential candidate genes for POI pathogenesis.

Xenobiotics, such as medicines and environmental chemicals, have a broad influence on the development of oocytes (Mark-Kappeler et al., 2011; Bhattacharya and Keating, 2012). Podophyllotoxin (POD) is a well characterized lignan, derivatives of which are widely used in clinical treatment due to their strong antitumor and antivirus activities (Ardalani et al., 2017; Zálešák et al., 2019). Exposure to POD can inhibit microtubule dynamics and lead to abnormal meiotic spindle formation, which influences oocyte development and maturation (Hu et al., 2018; Jiang et al., 2020). A research article in this Research Topic by Lu et al. illustrated that exposure to POD might disrupt protein synthesis, transport, degradation, and ATP production through its effects on the distribution and functions of organelles during mouse oocyte meiotic maturation.

Obesity can reduce ovarian and oocyte quality (e.g., spindle defects and chromosome misalignment) and ultimately impinge on female fertility. In addition, maternal obesity increases the level of oxidative stress in the ovarian environment (Silvestris et al., 2018; Igosheva et al., 2010; Jungheim et al., 2010). A research article in this Research Topic by Wen et al. reported that phycocyanin could recover the abnormal morphology of the spindle and reduce the accumulation of oxidative stress in oocytes. They further found that phycocyanin improves fertility by partially increasing ovarian and oocyte quality in obese female mice, providing a new potential strategy for clinically treating obesity-related infertility in women (Wen et al.).

*In vitro* maturation and other assisted reproductive technologies have been widely applied in the clinic in reproductive medicine centers to address the high rate of infertility worldwide (Cha and Chian, 1998; De Geyter, 2019). A research article in this Research Topic by Zhang et al. compared the global RNA transcription pattern of mouse oocytes from *in vitro* and *in vivo* maturation. They found that *in vitro* maturation resulted in metabolism and gene expression changes by environmental changes compared with *in vivo* matured oocytes.

## Conclusion

Over the past several years, significant progress has been made in understanding the biological functions and key events of meiosis. Despite these advances described above, many aspects of the biological processes remain poorly understood. Therefore, more comprehensive studies are required to further identify the molecular mechanisms of complex events that occur during meiosis, including regulation of the formation of programmed meiotic DSBs as well as the pairing, synapsis, recombination, and segregation of homologous chromosomes. We need to better understand chromosome interactions and chromosome movements, relying on continuing advancements in novel technologies and methods. Identification of the molecular basis of dynamic transcriptional regulation and protein post-translational modifications in meiosis remains a crucial endeavor. Understanding molecular mechanisms of meiosis could guide future research that is applied for the diagnosis and clinical treatment of infertility related to meiotic defects, and improve the health of our offspring.
